# Influence of Effective Laser Energy on the Structure and Mechanical Properties of Laser Melting Deposited Ti6Al4V Alloy

**DOI:** 10.3390/ma13040962

**Published:** 2020-02-21

**Authors:** Daojian Fu, Xiaoqiang Li, Minai Zhang, Min Wang, Zhen Zhang, Shengguan Qu

**Affiliations:** 1 Guangdong Key Laboratory for Advanced Metallic Materials Fabrication and Forming, National Engineering Research Center of Near-Net-Shape Forming for Metallic Materials, South China University of Technology, Guangzhou 510640, China; 2 Dongguan hyperpowder Co. Ltd., Dongguan 523808, China; 3 The Research and Development Center of Guangzhou Automobile Group Co. Ltd. (GAC R&D Center), Guangzhou 511434, China; 4 Automation Research Institute of China South Industries Group Corporation, Mianyang 621000, China

**Keywords:** additive manufacturing, laser melting deposition, laser energy, Ti6Al4V alloy, meso-structure, micro-structure, mechanical properties

## Abstract

The laser energy density (E_D_) is often utilized in many additive manufacturing (AM) processes studies to help researchers to further investigate the process-structure-property correlations of Ti6Al4V alloys. However, the reliability of the E_D_ is still questionable. In this work, a specific empirical calculation equation of the effective laser energy (E_e_), which is a dimensionless parameter in laser melting deposition (LMD) processing, was proposed based on the molten pool temperature. The linear regression results and the coefficient of determination prove the feasibility of the E_e_ equation, which indicates that E_e_ can more accurately reflect the energy-temperature correlations than the commonly used laser energy density (E_D_) equation. Additionally, Ti6Al4V components were fabricated by the LMD process with different E_e_ to investigate the influence of E_e_ on their structure and mechanical properties. Experimental results show that the detrimental columnar prior β meso-structure can be circumvented and the uniform α + β laths micro-structure can be obtained in LMD Ti6Al4V by a judicious combination of the process parameter (P = 2000 W, V = 12 mm/s, and F = 10.5 g/min) and E_e_ (7.98 × 10^5^) with excellent tensile strength (1006 ± 25 MPa) and elongation (14.9 ± 0.6%). Overall, the present work provides an empirical calculation equation to obtain a clearer understanding of the influence of different process parameters and indicates the possibility to fabricate the Ti6Al4V alloy with excellent mechanical properties by parameter optimization in the LMD process.

## 1. Introduction

Additive manufacturing (AM) of metallic components have aroused the excitement of considerable researchers due to its advantages such as near-net shape, short manufacturing cycle, high efficiency, and flexible manufacturing [1]. AM processes have also greatly promoted the application of some metallic materials that are difficult to fabricate via some conventional processes. In addition, the titanium alloy is undoubtedly one of the most representative metallic materials [2,3,4]. Ti6Al4V is considered to be the "main force" of the titanium alloys due to its excellent material properties. Therefore, it has received considerable attention. There are two common Ti6Al4V AM processes, which are known as the laser melting deposition (LMD) and selective laser melting (SLM), which are based on powder-feeding and powder-laying, respectively [5]. They all use laser as an energy source to melt the metallic powder. Therefore, laser energy, powder motion, and the dynamic interaction between them have significant impacts during manufacturing, which will, ultimately, determine the structure and properties of AM-built components [6,7].

Many researchers introduced a laser energy density (E_D_) concept [8,9,10,11] with the intention of describing the complex dynamic process under the AM process to help further investigate the process-structure-property correlations of AM-built Ti6Al4V alloys. The E_D_ describes a measurement of the averaged applied energy per volume of fabricated material, and the most commonly used energy density equation in SLM can be described by the following equation [9,12].

E_D_ = P/VHT, (1)

where E_D_ is the energy density, P is the laser power (W), V is the scanning speed (mm/s), H is the scanning pitch (mm), and T is the powder layer thickness (mm). Mierzejewska [9] investigated the effect of E_D_ on the porosity of SLM Ti6Al4V. He proposed that the optimal range of E_D_ is 78–127 J/mm^3^ to obtain a dense Ti6Al4V component. However, in relative research studies, different researchers have come up with different conclusions, which result in a huge optimal E_D_ range in SLM. The optimal E_D_ range is 120–180 J/mm^3^ and 120–195 J/mm^3^ reported by Han et al. [8] and Laquai et al. [13] respectively. Accordingly, Oliveira et al. [14] modified the E_D_ equation by introducing a dimensionless parameter β and defined E_D_ as:

E_D_ = βP/VHT (2)

where β is defined as the ratio between the powder size (g_s_) and the diameter of the heat source (d_HeatSource_): β = g_s_/d_HeatSource_. To evaluate the feasibility of the new equation, they recomputed the optimal E_D_ values for fabricating dense Ti6Al4V parts (>99%) based on key reference works, which were conducted in distinct experimental conditions. The results showed that, with the use of the dimensionless parameter β, the optimal E_D_ varies in a significantly narrower range (20–54 J/mm^3^) than its initial reports (33–293 J/mm^3^). Thus, they proposed that it is feasible to effectively compare the energy density in different SLM research studies by using the new equation. However, is the energy density a reliable parameter for materials synthesis by selective laser melting? Prashanth et al. [15] designed an interesting experiment to answer this question. They utilized the same powder materials and SLM equipment, and kept a constant energy density (55 J/mm^3^: calculated by Equation (1)) by only properly varying the laser power and the scanning speed to produce the bulk specimens. They found that the relative density of the fabricated specimens can range from 99.5% to 40%. The further investigation indicated that the E_D_ should not be considered as the only criterion in the optimization of process parameters during the SLM process. The same powder materials and SLM equipment ensure the consistent of the dimensionless parameter β in Equation (2). Thus, what they found also overturned the feasibility of Equation (2). The correlation between E_D_ and structures was also be investigated by others. Typically, AM-built Ti6Al4V contain reasonably α lamellae colony and epitaxially grown columnar prior β, which is considered as one of the drawbacks [16]. Kumar et al. [17] revealed the relationship between E_D_ and structures of SLM Ti6Al4V. They proposed that it is possible to tailor the detrimental columnar β by taking a suitable E_D_. Thus, a sample with excellent comprehensive mechanical performance can be manufactured. Overall, the reliability of the E_D_ Equations (1) and (2) is questionable. The equations have to be modified extensively to use the energy density concept for process parameters optimization effectively.

The energy density concept was also introduced in the LMD process and the equations got some improvement in relative research studies. Lia et al. [18] defined a relative equation about E_D_ for LMD research studies, as shown in Equation (3).

E_D_ = BP/Vd (3)

where B is the bulk absorption coefficient, and d is the diameter of the laser beam (mm). They tried to characterize the energy the powder actually absorbed by considering the absorption coefficient constant in the equation. Wu et al. [19] proposed a different thought. They found that simply halving both P and V and keeping other parameters do not present a similar Ti6Al4V micro-structure to that obtained at P and V, which is similar with what Prashanth et al. [15] found. Therefore, they proposed that E_D_ could be described in the following model.

E_D_ = Cα(P,V,F)P/VF (4)

where α is a function of P, V and F describe the effective energy absorbed by the powder, F is the powder feed rate (g/min), and C is a process constant. They proposed a new possibility with a new mathematical empirical model, but the investigation from Equation (4) is lacking.

The discussion of the energy density is the discussion of the thermal analysis [14]. However, the commonly used energy density Equations (1)–(3) cannot present this correlation very well. The most significant thermophysical characteristic during the manufacture is the temperature of the molten pool. There are only a few studies that use the molten pool temperature as one of the influencing factors to analyze the microstructural evolution. Yu et al. [20] decreased the heat accumulation effect in the LMD process by monitoring the pool size and temperature, and then varying the laser power in real-time to obtain a uniform Ti6Al4V component. The molten pool temperature should be a more reliable parameter to build the correlation of process-structure-property. In this study, a new concept of the effective laser energy was proposed, which is linked with the molten pool temperature measurement and can clearly reflect the influence of different process parameters. In addition, Ti6Al4V components were fabricated by the LMD process to further investigate the influence of the effective laser energy on their structure and mechanical properties.

## 2. Materials and Methods

### 2.1. Materials

**Figure 1:**
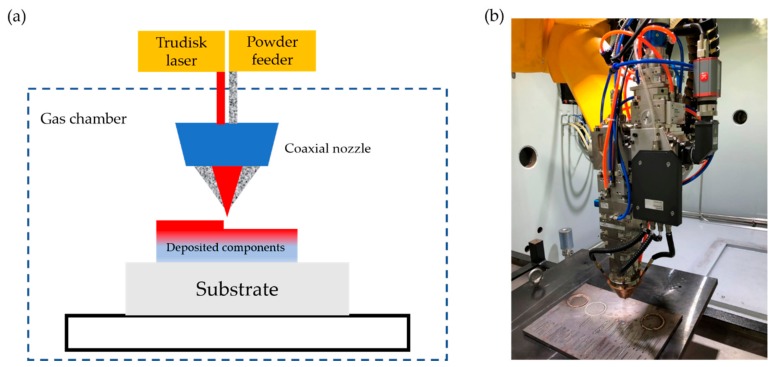
(**a**) The schematic and (**b**) the raw equipment of the laser melting deposition (LMD) process.

The manufacturing powder is a spherical Ti6Al4V powder with a particle diameter of 75–148 μm, which was fabricated by the plasma rotating electrode process (PREP). In addition, the chemical composition of it, which is examined by an inductively coupled plasma (ICP) device (720-OES, Varian, Atlanta, GA, USA) and the N/O Analyzer (TC600, Leco, St Joseph, MI, USA), is presented in Table 1. LMD was conducted in an LSF-I type laser melting deposition equipment (with a 4kW TRUMPF disk laser) self-developed by SWAI (LSF-I, SWAI, Mianyang, China). Figure 1a,b illustrates the schematic and the raw equipment, respectively. The molten pool temperature was monitored by an infrared thermometer (METIS M3, Sensor Therm, Sulzbach, Germany) with an effective temperature measurement range of 600–2300 °C.

### 2.2. Methods

**Figure 2:**
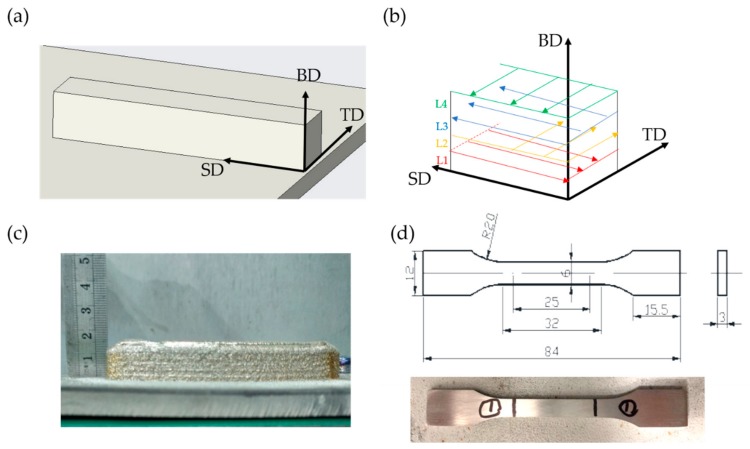
(**a**) The dimensional drawing of the deposited bulk; (**b**) The schematic of the scanning strategy; (**c**) The raw deposited component and (**d**) the tensile specimens.

The equation of the effective laser energy was defined by referring to the monitored molten pool temperature while depositing a 50 mm length single track. Afterward, Ti6Al4V bulk were fabricated in size of 110 × 20 × 20 mm^3^. The deposited substrate is an 8-mm thick rolled Ti6Al4V plate. In addition, the LMD parameters of the single track and Ti6Al4V bulk are shown in Table 2. It has to be realized that the lifting height of each layer (ΔZ) when depositing the bulk is determined by the process parameter in LMD, which means that different parameters will lead to different ΔZ. In the present work, a beam diameter of 3 mm was used in all research studies. Figure 2a,b show the dimensional drawing of the deposited bulk and the schematic of the scanning strategy, respectively (BD: building direction, SD: scanning direction, TD: traverse direction). The rotation of the scan direction between each successive layer is 90°, which is to ensure the uniformity of the heat input as well to circumvent porosity.

Samples with 10 × 10 × 10 mm^3^ were machined from the deposited bulks for density and microstructural investigations by wire cutting. The density of the samples was characterized by the Archimedes method when using an electronic balance (BSA224S, Sartorius, Göttingen, Germany). The micro-structure was characterized by optical microscopy (OM, DMi 5000, Leica, Wetzlar, Germany), X-ray diffraction (XRD, D8 Advance, Bruker Co., Bremen, Germany), scanning electron microscopy (SEM, Nova Nano 430, FEI, Hillsboro, OR, USA), electron back-scatter diffraction (EBSD), and transmission electron microscopy (TEM, JEOL2100F, JEOL, Tokyo, Japan). EBSD was conducted on the Nova Nano 430 equipped with an EBSD detector (Symmetry^®^, Oxford Instruments, Oxford, UK).

Three tensile specimens of each fabricated bulk with a dimension of 84 × 12 × 3 mm^3^ (as shown in Figure 2d) paralleled to SD were machined, and the tensile test was conducted using a testing machine (Instron 5569, Instron, Norwood, MA, USA) at room temperature with a rate of 0.033 mm/s. The rest of the specimens were utilized for a Micro-Vickers-hardness test on a hardness tester (DHV-1000Z, SCTMC, Shanghai, China) with a loading force of 500 g and a loading time of 15 s, and 10 points on the traverse plane were tested for each specimen.

## 3. Results and Discussion

### 3.1. The Effective Laser Energy

**Figure 3:**
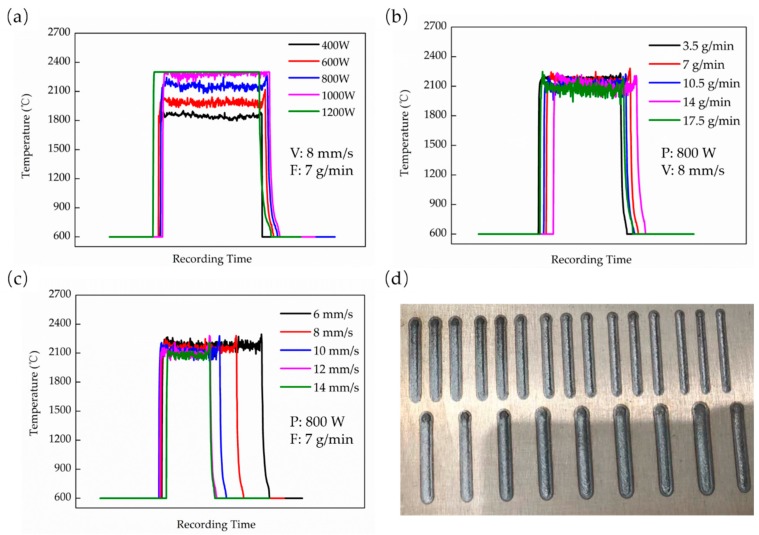
The effects of different process parameters: (**a**) laser power (P), (**b**) powder feed rate (F), and (**c**) scanning speed (V) on the molten pool temperature and (**d**) the deposited single track specimens.

The effect of different process parameters on the molten pool temperature was studied. Figure 3a–c shows the effect of a different laser power, powder feed rate, and scanning speed on the molten pool temperature, respectively. It indicates that the molten pool temperature increases as the laser power increases. The temperature can reach 1800 °C in 400 W and exceeds 2300 °C when P is above 1000 W (Figure 3a). However, the increase of scanning speed and powder feed rate will decrease the temperature even though the influence is slight. The temperature only varies around 2100 °C, as shown in Figure 3b,c. In general, the above equations can reflect these effects in some way. However, a significant difference can be seen from these results when the laser power, the scanning speed, and the powder feed rate vary in a close ratio. The magnitude of the temperature change is inconsistent. Moreover, it indicates that the influence of laser power is greater than the other two parameters. It is not a unique instance, but has its counterpart. Prashanth et al. [15] indicated that the laser power has a dictating influence on the porosity of the Ti6Al4V parts in their research of the reliability of E_D_. Therefore, it is very hard to maintain the same value of the temperature by simple proportional, changing-related parameters. That also explains what Wu et al. [19] reported that simply halving both P and V and keeping other parameters does not give a similar Ti6Al4V micro-structure to that obtained at P and V. Therefore, the energy density equations above seems to only reflect the positive and negative relationships between P, V, and F and the laser energy. These mathematical equations cannot be a reliable and accurate enough parameter for the effective comparison of different process parameters.

**Figure 4:**
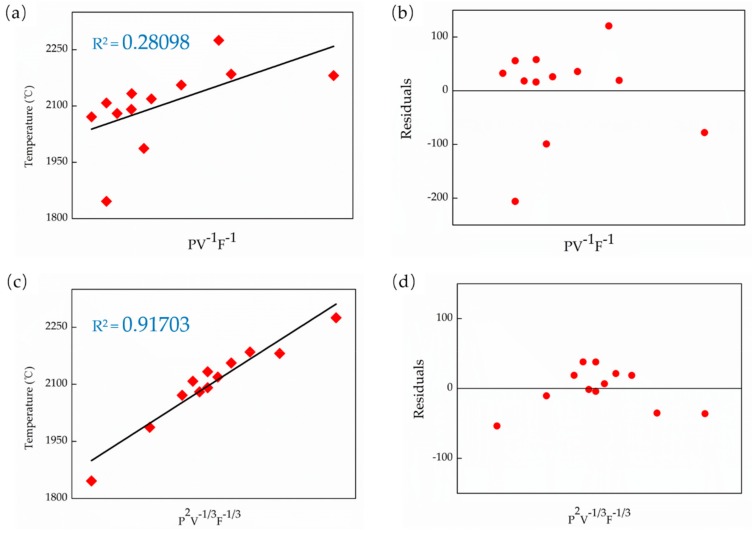
The results of linear regression: (**a**) The temperature versus PV^−1^F^−1^, (**b**) the residuals of PV^−1^F^−1^, (**c**) the temperature versus P^2^V^(−1/3)^F^(−1/3)^, and (**d**) the residuals of P^2^V^(−1/3)^F^(−1/3)^.

The energy density equations above (Equations (1)–(4)) are all empirical without a phenomenological physical demonstration [14]. These equations are only defined by considering the different process parameters as much as possible [15]. The lack of correlation with actual physical parameters may be the reason for their deficiency in practical applications. However, the α (P, V, F) function presented in Equation (4) still offers a possible direction to build a new empirical mathematical model. Defining a new empirical equation, which is associated with the actual physical parameter: molten pool temperature (T). To distinguish it from the energy density equation, call it “the effective laser energy” represented by E_e_. In addition, assuming T and E_e_ have a simple linear relationship in order to reflect the connection between them most intuitively, which can be described as T = aE_e_ + b, where a and b are constants. The mathematical model of E_e_ can be inspired by the energy density Equations (1)–(4), which means E_e_ can be defined as some combinations like E_e_ = P^δ^V^ε^F^λ^. From the research results on the effect of different process parameters on T and the previous E_D_ equations, it can be derived that the constants δ, ε, and λ should meet the following mathematical requirements: δ ≥ 1, 0 > ε ≥ −1 and 0 > λ ≥ −1. Then, using a linear regression method to determine the δ, ε, and λ. Additionally, to evaluate the feasibility of E_e_, the regression results conducted based on E_D_ was compared. The E_D_ equations can be recognized as PV^−1^F^−1^. Figure 4 shows the linear regression results between E and T. With our experimental results, it can be concluded that T is more proportional to P^2^V^(−1/3)^F^(−1/3)^ with a coefficient of determination R^2^ = 0.917, while the coefficient is only 0.281 when the linear regression was conducted based on PV^-1^F^-1^. Therefore, it indicates that the E_D_ cannot reflect the actual physical parameter very well, which lead to a deficiency on related thermal analysis. Moreover, we got a more detailed equation than Equation (4) by statistical analysis and linear regression. The effective laser energy (E_e_) can be described in the following equation.

E_e_ = P^2^/V^1/3^F^1/3^ (5)

There are two aspects that need to be noted about the E_e_ equation: (a) the beam diameter d is also an important factor to the energy equation, but the discussion of d is lacking in many research studies since the beam diameter is usually a fixed working parameter with no possible adjustment [14], which also happened in the present research, and (b) the E_e_ value can be recognized as a dimensionless parameter, which may not help to explain the specific physical phenomenon about the process but can be help to fully understand the influence of different process parameters. Such an analogous dimensionless parameter has already been used in the other AM study [21].

Four Ti6Al4V bulks with one bulk for each process parameter (as shown in Figure 2c) with different E_e_ were fabricated by the LMD process to investigate the influence of E_e_ on their structures and mechanical properties. Table 3 shows the specific process parameters of each component and their E_e_ value. The test samples are nominated T1, T2, T3, and T4 according to the value of E_e_ from low to high.

### 3.2. Meso-Structures and Micro-Structures

**Figure 5:**
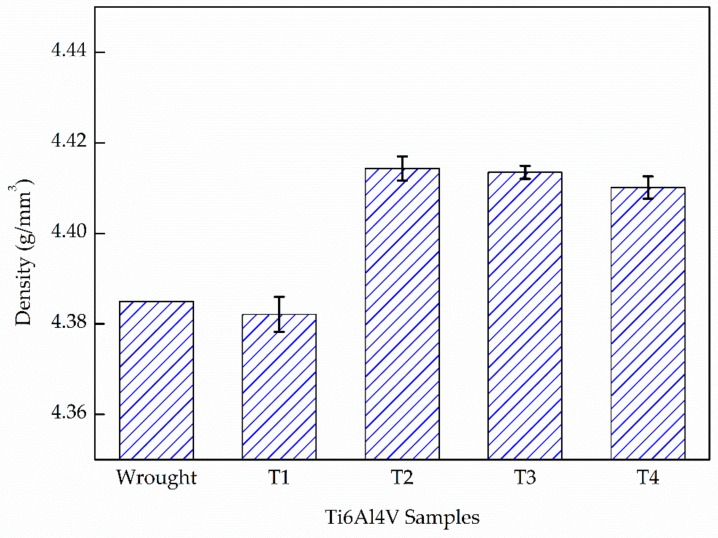
The density of the laser melting deposition-built samples compared with the wrought one.

Figure 5 shows the density results of the deposited Ti6Al4V samples compared with a wrought substrate. It shows that the wrought sample has a density around 4.38 g/mm^3^. Furthermore, the deposited T1 sample has the lowest density (4.37 g/mm^3^) and the lowest E_e_. As E_e_ increases, the density of the sample increases and stabilizes above 4.40 g/mm^3^ for the T2, T3, and T4 samples. It is worth noting that the substrate part shows a lower density than T2, T3, and T4. It may attribute to the residual casting defects in the wrought substrate (the substrate was fabricated by rolling from the cast slab). The increase in pores caused by overheating in higher energy reported by Mierzejewska [9] did not occur in the present work. Moreover, the relative density of these three deposited samples are all ≥99.60% (take a theoretical density of 4.43 g/mm^3^), which indicates that it is possible to fabricate a nearly-full density Ti6Al4V alloy via the LMD process with a sufficient E_e_.

#### 3.2.1. Meso-Structure

**Figure 6:**
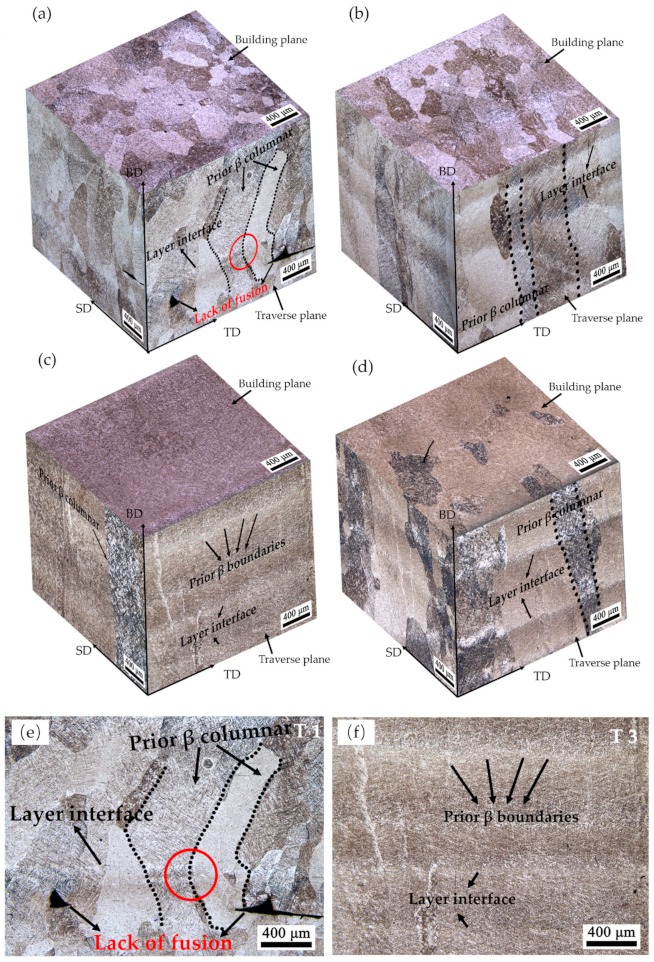
The 3D representative meso-structure of different LMD Ti6Al4V samples: (**a**) T1, (**b**) T2, (**c**) T3, and (**d**) T4. (**e**) The magnification image in the traverse plane of T1. (**f**) The magnification image in the traverse plane of T3.

Figure 6 shows the representative three-dimensional images of the meso-structure of the four samples observed by OM. A heterogeneous structure with different morphology can be seen in each sample. Most samples possess equiaxed grains in the building plane, and columnar grains in the scanning plane and traverse plane. This is similar with other reported results for AM-built Ti6Al4V [17,18,22]. However, some slight differences present in different samples.

For the T1 sample shown in Figure 6a, the equiaxed grains in the building plane are irregularly shaped, while the crystal boundaries are clearly visible and the grains are easy to distinguish. The detail in the traverse plane of T1 is shown in Figure 6e. Some long and deep pores in an irregular shape can be observed adjacent to successive layer interface. Therefore, these pores lead to a low density of T1. Defects in T1 are mainly attributed to a low laser power (P), a high powder feed rate (F), and a high layer thickness (ΔZ), which turn out to be the lowest E_e_. The E_e_ is too low to melt so much fed powder, while the large ΔZ of each layer track contributes to an incomplete re-melting of the upper surface of a previous layer. In addition, long columnar grains epitaxially grow through several layers. These columnar grains are considered to be prior β grains [23]. In most relative research studies, the prior β grains are predominantly near-parallel to the building direction [24] because the formation of the column is due to a high thermal gradient and a maximum heat flow vertical to the substrate, i.e., the building direction. However, the prior β grains in T1 are discontinuous and inclined, and the growth angle between each layer changed, as shown in the red circle area in Figure 6e, which has rarely been seen in relative research studies. Kumar et al. [17] proposed that the low laser energy will lead to the formation of molten zone boundaries, which results in discontinuous and jagged columnar grains. In addition, the inclined columnar β in T1 may attribute to the vertical direction not being the maximum heat flow direction during solidification. The incomplete melted powder and unfused pore defects will also conduct the input heat. Therefore, the maximum heat flow direction varies. Thus, the epitaxial growth angle varies in successive layers.

With the increase of E_e_, the long columnar prior β grains in T2 epitaxially grow to cross the entire plane (as shown in Figure 6b), which is completely parallel to the building direction. In addition, the grain boundaries are also easy to distinguish. No visible defects can be observed in T2, which is in line with its high density. The formation of this morphology is due to the sufficient melting of powder and the complete re-melting of the upper surface of a previous layer with a higher E_e_ (5.46 × 10^5^).

T3 presents a significantly different meso-structure morphology among all the samples shown in Figure 6c. The equiaxed grains in the building plane disappeared since the boundaries of the prior β grains are barely to be seen. In the scanning plane and traverse plane, only a few columnar crystals are still clearly visible. For most of the plane, as shown in Figure 6f, the columnar prior β grains are hard to distinguish and only some β grain boundaries can be faintly seen. This is a unique phenomenon that has almost never been seen in other studies. The columnar β grains are considered as the predominant structures in AM-built Ti6Al4V, and most researchers admit that the columnar β grains should be detrimental to the comprehensive properties of materials. Post-processing heat treatments were conducted to improve such a columnar structure to improve the detrimental influence in many research studies [17,25,26]. Yet, in the present research, T3 shows a unique meso-structure with almost no columnar grains. It indicates that it may be possible to fabricate an as-deposited Ti6Al4V components with an ideal structure without subsequent heat treatment. The specific reason for the formation of such a phenomenon will be discussed in Section 3.2.2 together with the formation of its micro-structure.

For T4 with the highest E_e_, as shown in Figure 6d, it is a common meso-structure, containing the irregularly equiaxed β grains in the building plane and columnar β grains in the scanning plane and traverse plane. While, in some areas, the prior β grain boundaries are also difficult to distinguish. The measurable columnar prior β grains in all samples have a close width between 200–500 μm. It is exactly the same as reported in many previous LMD Ti6Al4V studies [23,27].

#### 3.2.2. Micro-Structure

**Figure 7:**
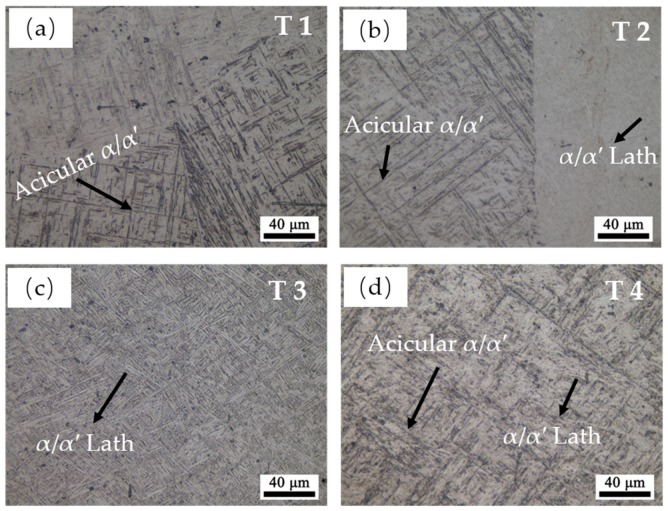
The magnification micro-structure image in the traverse plane of sample: (**a**) T1, (**b**) T2, (**c**) T3, and (**d**) T4.

The micro-structure magnification images in the traverse planes of the four samples are shown in Figure 7. A predominant acicular α/α′ phase can be seen in T1, which is shown in Figure 7a. These acicular α/α′ are either parallelly or perpendicularly distributed inside the prior β grains. The same acicular α/α′ can be observed in T2, while few α/α′ laths are also present in this sample, which can be barely found in T1. The predominant micro-structure in T3 is α/α′ laths. These α/α′ laths are also either parallelly or perpendicularly distributed, but it is more uniform and coarser than the acicular α/α′. Additionally, the acicular α/α′ is hard to be observed in T3. Yet, a number of acicular α/α′ and α/α′ laths can both be found in T4.

**Figure 8:**
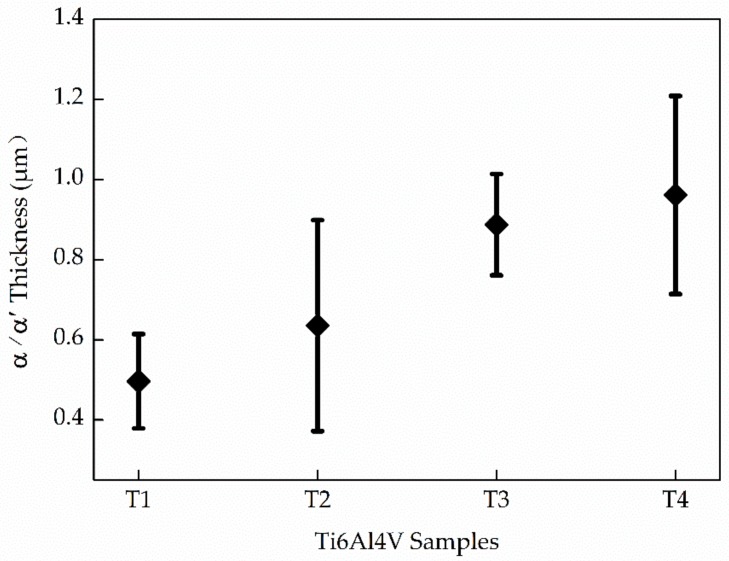
The thickness of the α/α’ phases of the four samples.

The formation mechanisms of the hexagonal close packed (HCP) structure α-Ti and martensitic α′ are significantly different. Therefore, it is necessary to further distinguish the α-Ti from martensitic α′ first. Some previous relative studies only simply distinguished these two phases by their different morphologies [28,29]. Figure 8 shows the thickness of the α/α′ phases of the four samples characterized by EBSD. The average thickness increases with the increase of E_e_. The specific mechanism behind will be discussed in Section 3.2.3. Additionally, the error bar of T2 and T4 is larger than T1 and T3, which is in keeping with the micro-structure features in T2 and T4 who both possess a certain number of acicular α/α′ and α/α′ laths. It should be noted that the highest gap between them is only 0.4 μm. Therefore, very similar morphology characteristics may not help to distinguish α from α′ correctly.

**Figure 9:**
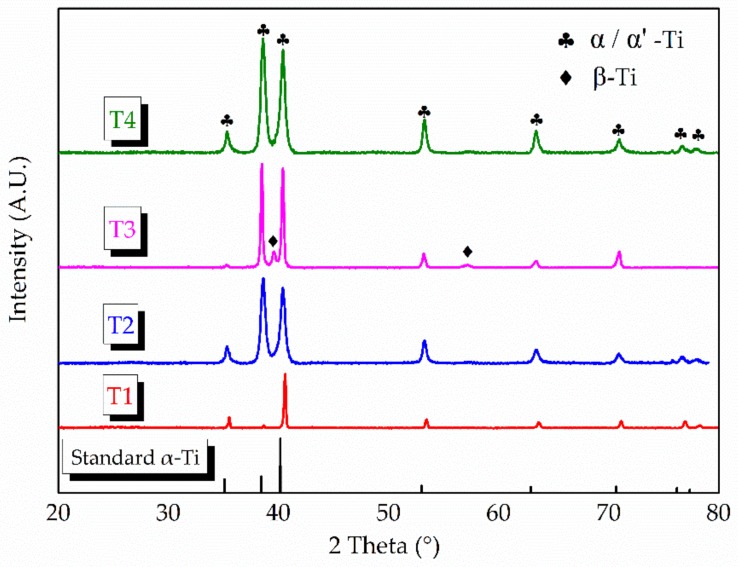
XRD patterns of the four samples compared with the standard hcp α-Ti peak.

XRD and TEM were utilized to further distinguish hcp α-Ti from martensitic α′. Figure 9 shows the XRD pattern result of the traverse plane. The standard hcp α-Ti (PDF: 44–1294) peaks are also presented. The β phase peaks only present in T3, which indicates a certain number of β-Ti present in this sample. It is worth noting that, in most previous AM-built Ti6Al4V research studies [2,7], the β phase only presents after post processing heat treatment. All the α phase peaks in T1 show a slight shift to the higher 2 theta angles. The same situation happens on the other samples when the scan angles are beyond 50°. Qiu et al. [24] reported a similar phenomenon, and, therefore, deduced the presence of martensite α′ phase in their research, for the slight shifts in XRD patterns often mean the formation of supersaturated solid solution. However, these slight shifts may not be convincing enough for distinguishing α from α′. The martensitic α′ phase also has an hcp crystal structure and the c/a ratios of α and α′ are very close (the c/a value of α′ is 1.589 while the c/a value of α is 1.59–1.60) [30]. Furthermore, TEM images and phase composition analysis could help to resolve this because V is known as a β phase-stable element [31], and α′ is known as a non-equilibrium phase that may contain a higher content of V compared to α.

**Figure 10:**
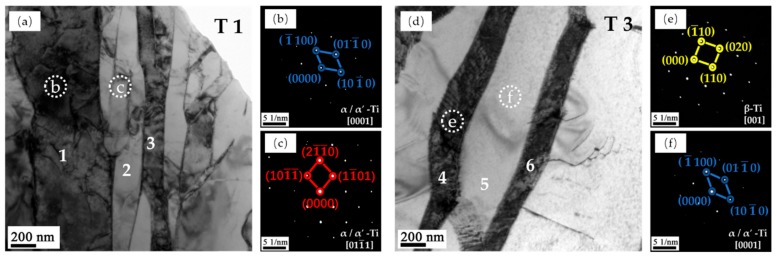
The TEM images of T1 and T3 samples showing two different types of laths: (**a**)T1, (**b**) SADP image of marked point b, (**c**) SADP image of marked point c, (**d**) T3, and (**e**) SADP image of marked point e. (**f**) SADP image of marked point f.

The TEM images of T1 and T3 are shown in Figure 10. Two different types of laths can be observed. The laths in T1 are rugged and jagged, while T3 presents a smoother one, which indicates a structure distortion that may present in T1’s micro-structure. Moreover, although dark and bright contrast laths appear in both samples, there are significant differences between them. Figure 10b–f shows the selected area diffraction patterns (SADP) corresponding to the areas as indicated by the white dotted circle, respectively. It indicates that the dark contrast phase in T1 presents an hcp α/α′-Ti structure, while the dark laths in T3 are the β-Ti structure. In addition, the bright contrast laths in both samples are the α/α′-Ti phase. TEM-EDS (energy dispersive spectrometer) was utilized to analyse the elemental composition of points 1–6 in Figure 10a,d, and the results are presented in Table 4. The β-Ti phase (points 4 and 6) contains much more V, which verifies that V is a β phase-stable element. However, the V content of the α/α′-Ti phase is relatively different in the two samples. The α/α′-Ti phase in T1 contains a higher V than T3, and the elemental composition of T1 is closer to that of the original Ti6Al4V powder, which indicates that the cooling rate is too fast to allow V to diffuse in T1. Therefore, the α/α′-Ti phase in T1 could be inferred to be a martensitic α′-Ti phase. In addition, the micro-structure in T3 could be inferred to be the α + β laths Widmanstätten structure.

**Figure 11:**
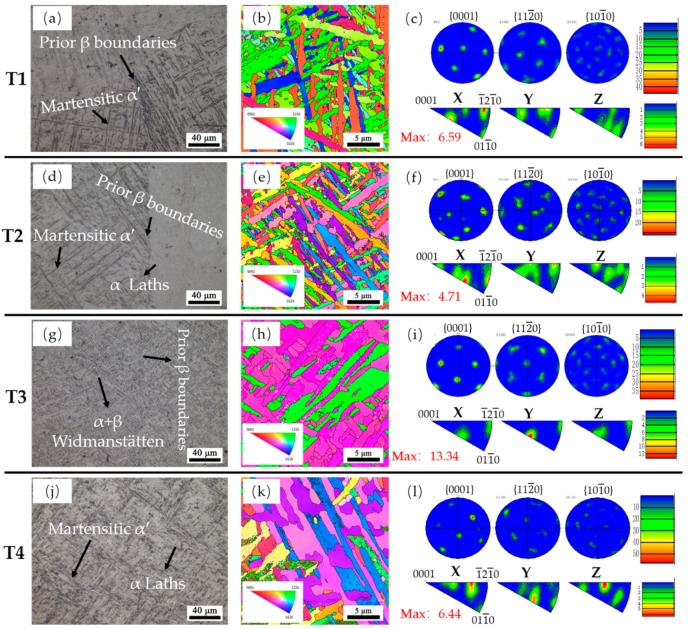
The specific micro-structures of the four samples and the texture examination results. (**a,d,g,j**) micro-structure features for T1, T2, T3, and T4, respectively. (**b,e,h,k**) The EBSD inverse pole figure maps and (**c,f,i,l**) corresponding pole and inverse pole images.

Figure 11 shows the specific micro-structures of the four samples and the texture results examined by EBSD. The predominant micro-structure in T1 (as shown in Figure 11a) is the martensitic α′-Ti phase for a low E_e_. With the increase of E_e_, the micro-structure varies to a α′ + α phase in T2 (Figure 11d). For T3 (Figure 11g), a much higher E_e_ and the predominant micro-structure turns into α + β laths Widmanstätten structure. In addition, the predominant micro-structure of T4 (Figure 11j) is also an α′ + α phase like T2. The α/α′ phases of the samples often present different morphology or orientation within different prior β grains, as shown in Figure 11a,d. T3 presents a similar morphology and orientation on different sides of the prior β boundaries (Figure 11g). That is why the columnar prior β grains are hard to be distinguished in T3’s meso-structure. 

The texture of the α/α′ phase in the four samples shown in Figure 11 may help to infer the forming mechanisms of these micro-structures. T1, T2, and T4 present a random texture with relatively weak intensity. It is a common result for AM-built Ti6Al4V components because the transformation of β to α follows Burger’s orientation relationship strictly, which means each β phase can transform into 12 different α orientations [32]. Sridharan et al. [33] reported the texture evolution of Ti6Al4V during the LMD process. They proposed that the initial random textures will transform to a strong basal texture as a result of the multiple thermal cycles. In present work, T3 presents a more uniform texture, as shown in the inverse pole figure (IPF) maps with the highest intensity of 13.34. It indicates that the α/α′ phase in T3 must have went through a uniform and consistent phase transformation because of the thermal influence of subsequent depositions.

**Figure 12:**
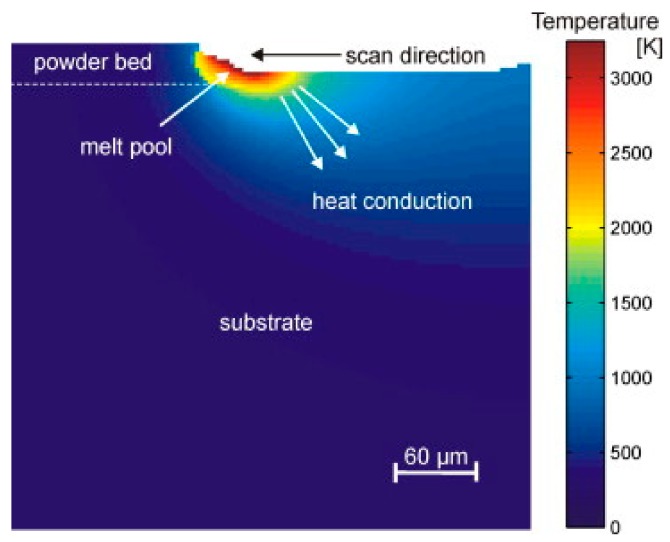
The thermal schematic of the AM process [10,34].

The forming mechanism of AM-built component’s micro-structure is mainly affected by the following two methods, as shown in Figure 12 [10,34]: (1) the cooling rate of the deposited molten pool, and (2) the thermal influence of the deposited layer on the previous layers. Such an effect is like the heat treatment effect of annealing or tempering (also called intensified intrinsic heat-treatments). It has been well reported that a slow cooling rate (less than 3.5 °C/s) from the β phase transition temperature will lead to the decomposition of β to α + β in the Ti6Al4V alloy, but the AM process has extremely high cooling rates, which means only martensitic α′ can present. That is why the β phase only presents after subsequent heat treatment in most previous AM-built Ti6Al4V research studies [2,7]. Therefore, the formation of the unprecedented micro-structure in the present work must have a further reason.

#### 3.2.3. Structures Forming Mechanism

**Figure 13:**
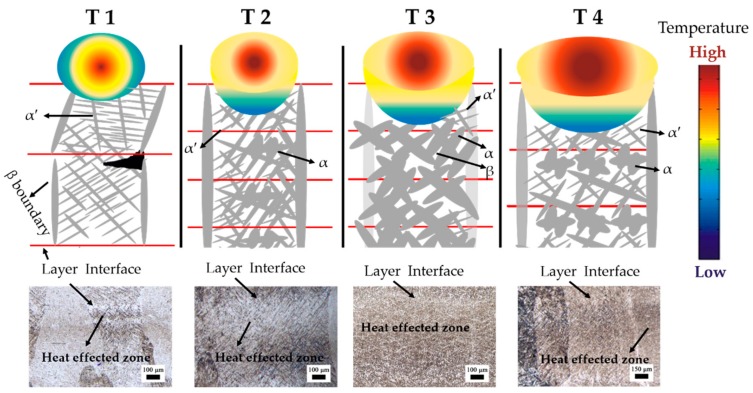
The schematic micro-structure forming mechanism of present laser melting deposition (LMD) Ti6Al4V samples.

The schematic of the micro-structure forming mechanism of the four samples is shown in Figure 13. The lowest input E_e_ (2.07 × 10^5^) value of T1 provoke the highest thermal gradient during manufacturing. A high thermal gradient leads to a fast solidification rate. As a result, acicular martensitic α′ begins to present. Meanwhile, such a low E_e_ means the re-melting effect of the deposited layer on the previous layer will be limited in a very small area, so only very little α′ to α transformation can be conducted in T1. Eventually, the acicular martensitic α′ become the predominant micro-structure in T1. Overall, the forming mechanism (1) is the dominant one for T1’s micro-structure with the lowest E_e_.

With the increase of E_e_ (5.46 × 10^5^), the thermal gradient in T2 is decreased. Yet its cooling rate is still high, and acicular martensitic α′ will still be present during solidification. However, the re-melting effect in T2 is stronger than T1. Higher heat input and a larger effected zone make much more α′ transform to α. However, the thermal energy is unable to affect the entire previous layer, so there is still considerable untransformed acicular martensitic α′ remaining. Therefore, the acicular martensitic α′ and α laths can both be observed in T2. The mechanism (1) and (2) jointly work in T2. Moreover, the sufficient re-melting of the upper surface of a previous layer also enables columnar crystals to maintain perpendicular epitaxial growth.

The E_e_ value of T3 is much higher (7.98 × 10^5^), which provoke a slower cooling rate, and the slightly coarser acicular martensitic α′ is present. The re-melting effect now is further stronger and the heat effected zone can almost involve the entire previous layer. Therefore, considerable α′ transfers to α, and the β phase begin precipitating between adjacent lamellar α phases. Even the prior β grain boundaries of α phases begin transferring. Afterward, only a few α′ phase remains. The predominant micro-structure in T3 becomes an α + β Widmanstätten structure. Thus, the forming mechanism (2) should be the dominant one in T3.

Yet, the higher E_e_ is not better. When E_e_ comes to the highest one (9.13 × 10^5^) in T4, while its predominant micro-structure is not the α + β Widmanstätten structure but a α′ + α laths structure. The process parameters of T4 is a 2000 W laser power like T3. However, the scanning speed of T4 is slower (as shown in Table 3), which lead to the highest E_e_ value. Decreasing the scanning speed will also increase the layer thickness, which has been reported by relative LMD research studies [35]. Therefore, although T4 has the highest E_e_, its layer thickness also increases, and the heat affected zone cannot involve the entire previous layer at this time. A certain amount of α′ decompose to α + β, while a certain amount of the α′ phase is also retained. Thus, the predominant micro-structure in T4 is α + α′ laths.

The non-equilibrium α′ phase, as the most common micro-structure of AM-built Ti6Al4V, is considered detrimental to the ductility of deposited components. Thus, considerable efforts were conducted to utilize a subsequent heat treatment to improve the deposited micro-structure [26,36]. However, such an extra step makes AM lose its advantages of rapid manufacturing. Barriobero et al. [37] utilized an intensified intrinsic heat-treatment method by using a very tight hatch distance to increase laser exposure time to obtain a stable α + β micro-structure of SLM Ti6Al4V. However, a great number of untransformed α′ is still presented in their research. In the present work, a Ti6Al4V component containing stable and uniform α + β laths micro-structure is also fabricated by analogous intensified intrinsic heat-treatment. It indicates possible directions to provoke the non-equilibrium α′ decomposition to stable α + β by a judicious combination of the process parameter (P = 2000 W, V = 12 mm/s, and F = 10.5 g/min) and E_e_ (7.98 × 10^5^) in LMD Ti6Al4V.

### 3.3. Mechanical Properties

**Figure 14:**
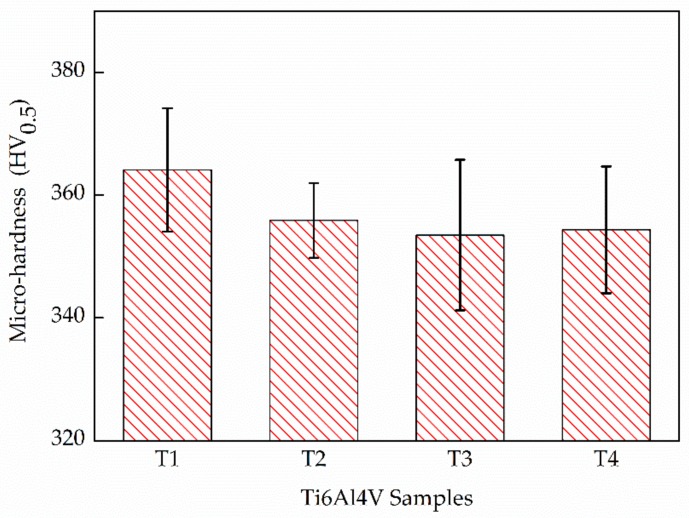
The micro-hardness (HV_0.5_) of LMD Ti6Al4V samples.

Figure 14 shows the micro-hardness results of the four deposited components. The as-deposited Ti6Al4V components all have a superior micro-hardness exceeding 350 HV. T1 presents the highest micro-hardness value with 360 ± 10 HV due to the predominant α′ micro-structure. In addition, T3 with the predominant α + β micro-structure presents the lowest micro-hardness value of 353 ± 12 HV. The left two samples have an approximative micro-hardness due to their similar micro-structures. It is worth noting that the error bars of the results show a large range, i.e., 20 HV. It indicates that more than one phase is presented in all test samples, i.e., the α′ + α + β phases.

**Figure 15:**
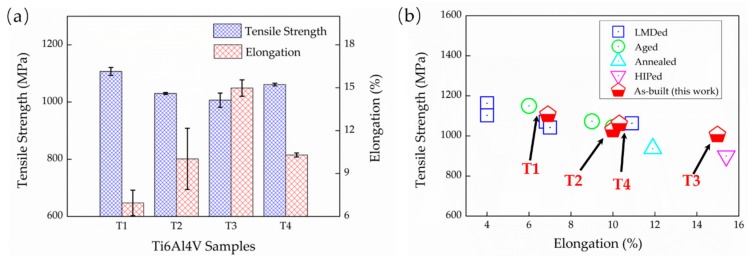
(**a**) Histogram of ultimate tensile strengths (σ_U_) and elongation (e_f_) in the present work, and (**b**) tensile properties comparation between the LMD Ti6Al4V reported by Liu et al [4] and present work.

The tensile properties of the as-deposited Ti6Al4V samples, including the ultimate tensile strengths (σ_U_) and the elongation (e_f_), are presented as a histogram in Figure 15. The as-deposited samples all possess superior ultimate strengths, exceeding 1000 MPa, which is higher than the strength requirement of wrought Ti6Al4V. This is because of the advantages of fine micro-structures obtained by additive manufacturing, which lead to a fine grain strengthening. Specifically, T1 presents the highest σ_U_ but the lowest e_f_, with 1107 ± 13 MPa and 6.9 ± 0.9%, respectively. The other three samples show a close strength while T3 presents the highest e_f_ of 14.9 ± 0.6% compared to a ~10% e_f_ of T2 and T4. An excellent ductility is presented in T3, which is rather rare in the AM-built Ti6Al4V. Figure 15b compares the properties of various LMD Ti6Al4V. It shows that T1 presents similar mechanical properties to the other LMD components reported by Liu et al [4], and the mechanical properties of T2 and T4 are closer to the aged LMD Ti6Al4V. The highlight is that T3 exhibits an excellent mechanical property, which is even better than the hot isostatic pressing (HIP) treated LMD Ti6Al4V for a comparable ductility but a higher strength. It means that it is possible to obtain an ideal Ti6Al4V component directly through LMD without any subsequent heat treatment. Additionally, such enhancements can be achieved through an intensified intrinsic heat-treatments effects with a judicious combination of E_e_ and LMD process parameters.

**Figure 16:**
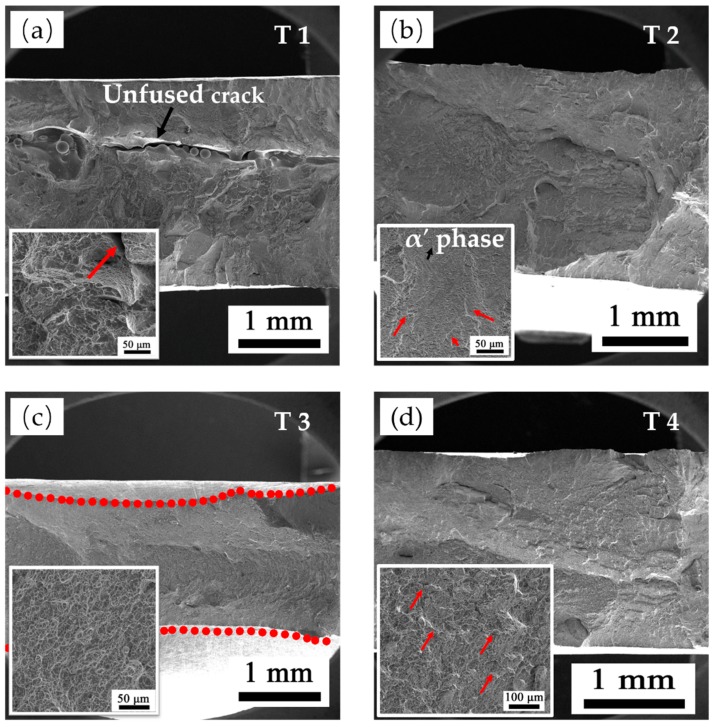
The tensile fractographies of LMD Ti6Al4V samples: (**a**) T1, (**b**) T2, (**c**) T3, and (**d**) T4.

Figure 16 shows the tensile fracture SEM images. A long unfused crack crossing the entire fracture plane can be observed in T1, which contains lots of un-melted powders. Though small dimples can be found in the magnification image, a tear edge with deep cracks is also displayed as indicated by the red arrow, which indicates a poor ductility in T1. T2 and T4 have a similar tensile feature. Their fracture surfaces are relatively corrugated with the distribution of small river-patterned cracks. The river-patterned cracks occur along the columnar prior β boundaries, and the α grains can be observed clearly within β grains in T2. Furthermore, small dimples are also presented in both samples, which indicates a general ductile-type fracture. T3 presents a unique fracture with a clear necking phenomenon, as indicated by the red dotted lines in Figure 16c and a relatively flat surface decorated with larger dimples, which both indicate an excellent ductility.

**Figure 17:**
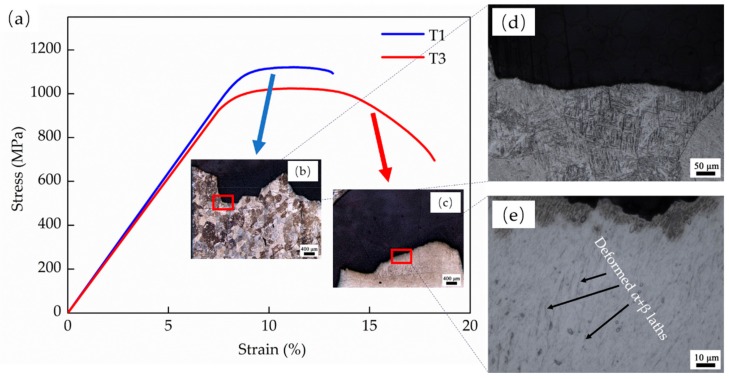
The tensile curves of T1 and T3 and the corresponding cross-sectional metallographic structure of fracture: (**a**) the strain-stress curves; (**b,d**) the metallographic structure of T1, and (**c,e**) the metallographic structure of T3.

To further understand the fracture mechanism, the fracture metallographic structures on the building plane side of T1 and T3 are presented in Figure 17 with the corresponding tensile curves. Cracks propagate through the equiaxed prior β crystal in T1, which exhibits a typical trans-granular fracture. The magnification image also shows that the crack passes directly through considerable acicular martensitic α′ phases, and these acicular phases have almost no deformation, who are still distributed in the prior β in parallel or perpendicular. While, the α + β laths in T3 suffered severe deformation, and the laths turned to be parallel to the direction of the applied tensile strain. Therefore, the high strength but low ductility in T1 should be attributed to a trans-granular fracture mode and considerable fine martensitic α′, which leads to fine grain strengthening and solution strengthening. This effectively prevents dislocation movements. Furthermore, the reason of the excellent ductility of T3 is its unique α + β laths structure, which is easy to deform and allows massive dislocations propagation. For the high strength of T3, there are two specific reasons, (a) the sufficient E_e_ fabricates a strong metallurgical bonding, and (b) the few retained martensitic α′ in T3 acts like a strengthening phase. The heterogeneous structures may lead to anisotropic properties in AM-built Ti6Al4V components, where the relative study is ongoing.

## 4. Conclusions

Based on the molten pool temperature, a specific calculation equation of the effective laser energy (E_e_) absorbed by the powder during LMD processing was explored, and the influence of E_e_ on the structure and mechanical properties of the LMD Ti6Al4V was further studied. Some conclusions can be drawn as follows.

(1) By comparing and analyzing the temperature data of the molten pool, we find that the commonly used energy density equation in the AM process does not reflect the thermal phenomenon very well. Therefore, a dimensionless parameter, which is the effective laser energy (E_e_), in the LMD process is proposed as E_e_ = P^2^V^(−1/3)^F^(−1/3)^, and the corresponding linear regression results showed that the coefficient of determination is 0.917, which is three times as that of the commonly used energy density equation.

(2) Based on the defined formula analysis, a low E_e_ (2.07 × 10^5^) will lead to the unfused pore defects and inclined columnar prior β grains. As the E_e_ increases, the near-full dense Ti6Al4V bulks can be obtained. Moreover, the common columnar prior β grain meso-structure in most AM-built Ti6Al4V components can be circumvented by a judicious combination of a process parameter (P = 2000 W, V = 12 mm/s and F = 10.5 g/min) and E_e_ (7.98 × 10^5^).

(3) For the micro-structure of deposited Ti6Al4V alloys: As the E_e_ increases, so does the transformation of α′ to α in the heat effected zone. The heat effected zone can nearly involve the entire previous deposited layer for a judicious combination of the process parameter (P = 2000 W, V = 12 mm/s, and F = 10.5 g/min) and E_e_ (7.98 × 10^5^). In addition, the uniform α + β laths micro-structure can be obtained without any post-treatment.

(4) The LMD Ti6Al4V alloys exhibit dramatic mechanical properties compared to the conventional process. Martensite α′ and defects obtained at low E_e_ will cause the components to have high strength (1107 ± 13 MPa) but poor ductility (6.9 ± 0.9%). However, the α + β laths with few α′ micro-structures obtained at a sufficient E_e_ results in an excellent comprehensive mechanical property, i.e., high strength (1006 ± 25 MPa) and ductility (14.9 ± 0.6%), which is even better than the HIP treated LMD Ti6Al4V.

## Figures and Tables

**Table 1:** Chemical composition and material characteristics of used powder.

Element	Ti	Al	V	Fe	O	N	C
wt.%	Bal.	6.53	4.13	0.25	0.08	0.04	0.01

**Table 2:** Laser melting deposition (LMD) processing parameters of the Ti6Al4V single track and bulk.

Parameter	Single Track	Ti6Al4V Bulk
Laser power/P (kW)	0.2–1	1–2
Scanning speed/V (mm/s)	6–14	8–12
Powder feed rate/F (g/min)	3.5–14	7–14
Beam diameter/d (mm)	3	3
Layer thickness/ΔZ (mm)	-	0.6–1.5

**Table 3:** The specific laser melting deposition (LMD) process parameters of Ti6Al4V bulks and their E_e_ value with a beam diameter of 3 mm.

Nomenclature	P (W)	V (mm/s)	F (g/min)	ΔZ (mm)	E_e_
T1	1000	8	14	1.58	2.07 × 10^5^
T2	1500	10	7	0.60	5.46 × 10^5^
T3	2000	12	10.50	0.63	7.98 × 10^5^
T4	2000	8	10.50	1.11	9.13 × 10^5^

**Table 4:** Representative elemental distributions for T1 and T3 samples analyzed by TEM-EDS.

Spot	Elements (wt.%)			
	Ti	Al	V	Fe
1	89.93	6.70	3.02	0.33
2	90.39	5.90	3.53	0.16
3	90.34	6.02	3.49	0.13
4	77.68	5.31	15.69	1.31
5	91.57	6.46	1.62	0.33
6	62.65	3.98	31.87	1.48
